# A prospective, randomized trial of the effect of buprenorphine continuation versus dose reduction on pain control and post-operative opioid use

**DOI:** 10.1097/MD.0000000000032309

**Published:** 2022-12-23

**Authors:** Aurora Quaye, Kristen Silvia, Janelle Richard, Yussr Ibrahim, Wendy Y. Craig, Clifford Rosen

**Affiliations:** a Department of Anesthesiology and Perioperative Medicine, Maine Medical Center, Portland, ME; b Spectrum Healthcare Partners, South Portland, ME; c Division of Addiction Medicine, Maine Medical Center, Portland, ME; d MaineHealth Institute for Research, Scarborough, ME.

**Keywords:** buprenorphine, opioid use disorder, protocol, substance use disorder

## Abstract

An increasing number of individuals are taking buprenorphine for management of opioid use disorder (OUD). Pain control can be challenging when these patients develop acute pain requiring supplemental analgesia. Buprenorphine’s pharmacokinetic profile can render supplemental opioid-based analgesia ineffective. There is limited guidance on the optimal management of buprenorphine when acute pain is anticipated. Although there is growing acceptance that the risk of OUD relapse with buprenorphine discontinuation overshadows the risks of increased opioid utilization and difficult pain control with buprenorphine continuation, perioperative courses comparing buprenorphine dose reduction and full dose buprenorphine continuation have yet to be investigated. Here, we describe the protocol for our randomized controlled, prospective trial investigating the effect of buprenorphine continuation compared to buprenorphine dose reduction on pain control, post-operative opioid use, and OUD symptom management in patients on buprenorphine scheduled for elective surgery.

**Methods and analysis:**

This is a single institution, randomized trial that aims to enroll 80 adults using 12 mg buprenorphine or greater for treatment of OUD, scheduled for elective surgery. Participants will be randomly assigned to receive 8mg of buprenorphine on the day of surgery onwards until postsurgical pain subsides or to have their buprenorphine formulation continued at full dose perioperatively. Primary outcome will be a clinically significant difference in pain scores 24 hours following surgery. Secondary outcomes will be opioid consumption at 24, 48, and 72 hours postoperatively, opioid dispensing up to 30 days following surgery, changes in mood and withdrawal symptoms, opioid cravings, relapse of opioid misuse, and continued use of buprenorphine treatment postoperatively.

## 1. Introduction

Opioid use disorder (OUD) is a chronic neurobehavioral disease characterized by continued opioid use despite the desire to quit, often punctuated by cycles of remission and opioid use relapse.^[1]^ OUD is associated with high rates of medical co-morbidity and life expectancy is 2 decades shorter when compared to those without OUD.^[2]^ In the past 20 years, opioid related deaths have risen exponentially.^[3–5]^ Unfortunately, despite our national crisis, opioids remain prominent in perioperative analgesic regimens for the millions of surgeries and procedures performed each year in the United States.

Buprenorphine is a high-affinity, partial mu opioid receptor agonist that is effective, life-saving opioid maintenance treatment for individuals with OUD. Multiple studies have shown that buprenorphine improves treatment retention, decreases illicit opioid use by reducing cravings and the withdrawal symptoms that occur between episodes of opioid misuse, and improves survival.^[6–8]^ The optimal duration of buprenorphine maintenance has not been established and relapse rates can be high when it is discontinued. A multi-site study sponsored by the National Institute on Drug Abuse reported relapse rates approaching 90% following the premature discontinuation of opioid maintenance treatment.^[8]^

Due to the pharmacokinetics of buprenorphine, managing acute post-surgical pain can be challenging, especially when full mu opioid receptor agonists are used concomitantly. Currently, there are no definitive recommendations to guide the perioperative management of buprenorphine. The Substance Abuse and Mental Health Services Administration (SAMHSA) recommends discontinuation of buprenorphine as an option prior to surgery- with full opioid agonist replacement for withdrawal prevention- to facilitate postoperative analgesia.^[9]^ However, SAMHSA states as an advisory warning that this can introduce significant risks to an individual’s substance use recovery, including cravings and relapse. Conversely, it also recommends full dose buprenorphine continuation as a treatment option, warning that the risks associated with this management strategy include ineffective analgesia necessitating escalating opioid administration. Because of the significant risks of relapse, buprenorphine continuation with and without dose reduction, have been suggested as strategies for perioperative pain management.^[10–12]^

Preliminary clinical observations support that buprenorphine continuation at low analgesic doses (8 mg) can adequately facilitate postoperative pain management without interrupting OUD treatment.^[13,14]^ However, the dose at which buprenorphine should be continued has not been established. Recent reviews contend that buprenorphine should be continued without dose adjustment perioperatively; however, this conclusion is based on professional opinion rather than on the results of clinical studies.^[10,15]^ In contrast, others report that OUD patients experience poorly controlled postoperative pain when maintained on their full treatment dose of buprenorphine and that this strategy results in increased opioid utilization and the disruption of hospital course.^[16,17]^ No studies have performed a direct comparison of continuing buprenorphine treatment at full versus low doses during the perioperative period.

### 1.1. Objectives

The primary objective of this study is to compare the efficacy of low dose buprenorphine continuation (STD-BUP) to full dose continuation (FULL-BUP) during post-operative pain control management for adults maintained on buprenorphine for OUD. The primary outcome is pain, measured by a numeric rating scale (NRS) of 0 to 10, a standard tool used in comparing analgesic efficacy between distinct treatment interventions.^[18]^

Our secondary objectives will be to compare postoperative opioid consumption (excluding buprenorphine formulations) in adults maintained on buprenorphine for OUD, measured as morphine milligram equivalents (MME) at 24, 48 and 72 hours postoperatively. Additionally, we will identify if OUD symptom severity and opioid utilization in the month following surgery is influenced by buprenorphine management strategy. We will investigate this by comparing OUD symptom severity and changes in mood and withdrawal symptoms, measured preoperatively and 30 days postoperatively, using the Patient Health Questionnaire-9 (PHQ-9), Current Opioid Misuse Measure (COMM), and Opioid Craving Scale (OCS) instruments. We will also compare the number of opioid prescriptions and quantity (in MME) of opioid medications dispensed, including buprenorphine dispensing, up to 30 days following surgery. To investigate OUD treatment retention and to identify prevalence of missed treatment appointments or opioid relapse, we will contact each enrolled subject’s buprenorphine provider 1 month following surgery.

The overall goal of our study is to inform the development of guidelines for the perioperative management of patients on buprenorphine. We anticipate that the findings of this study will contribute to helping patients and providers establish informed treatment decisions regarding optimal buprenorphine management practices.

## 2. Methods and analysis

### 2.1. Study design

This study is a randomized single blinded controlled trial that was reviewed and approved by the MaineHealth Institutional Review Board (IRB). This study protocol complies with the Recommendations for Interventional Trials (SPIRIT) 2013 statement guidelines and was designed according to the SPIRIT checklist.^[19]^

### 2.2. Study population

Adults aged 18 to 75 years old, currently taking buprenorphine formulations equivalent to 12 mg or greater for at least the prior 30 days for treatment of OUD (as defined by the Diagnostic and Statistical Manual of Mental Disorders-5 criteria), and scheduled for elective surgery associated with NRS ≥ 4 on postoperative day (POD)-1 at our academic hospital will be invited to participate in this study. The clinicaltrials.gov (NCT04981678) listing provides details regarding study locations. Exclusion criteria will include patients that are using buprenorphine formulations for pain management and those with a current major medical illness that could limit the ability to utilize non-opioid analgesic medications, such acetaminophen and ibuprofen. A full list of our eligibility criteria is shown in Table 1. For true practice generalizability on the association between buprenorphine management and postsurgical pain control, we will include surgical procedures based on average pain intensity POD 1, as opposed to limiting our criteria to a particular surgical type (Supplement 1, Supplemental Digital Content, http://links.lww.com/MD/I129). Our pragmatic design ensures that both dropout will be low, and that our study will evaluate perioperative buprenorphine management under usual clinical care conditions.

**Table 1:** Eligibility criteria.

Inclusion criteria
Adults aged 18–75 yr
ASA health classification I–III
Taking buprenorphine formulations equivalent to 12 mg or greater for at least the prior 30 d for treatment of OUD (as defined by the DSM-V criteria)
Scheduled for elective surgery at Maine Medical Center where moderate-severe pain is expected on POD1) (see Supplement 1, Supplemental Digital Content for list of surgeries)
Exclusion criteria
ASA classification 4–5
Buprenorphine use prescribed for chronic pain
Pregnancy or trying to become pregnant
Current uncontrolled major medical illness, such as cancer and end-stage organ failure
History of intellectual disability or other severe developmental disorder
Current diagnosis of delirium or dementia

ASA = american society of anesthesia, DSM-V = diagnostic and statistical manual of mental disorders-5, OUD = opioid use disorder, POD = postoperative day.

### 2.3. Participant recruitment and enrollment

Eligible participants will be notified about the study when they present for their pre-operative assessment prior to surgery. The majority of patients scheduled for surgery receive pre-surgical consultation within 2 months prior to their procedure. Individuals who express interest in the study will be scheduled for an enrollment visit where a study physician, or their delegate, will review the study procedures, providing them with the consent form, and answering any questions they have about the study. The consent form is available as supplemental information (Supplement 2, Supplemental Digital Content, http://links.lww.com/MD/I130). Participants that choose to participate in the study and consent to enrollment will be randomized to the treatment arms of FULL-BUP versus STD-BUP. Because consent needs to be given prior to day of surgery in order to randomize patients for this trial, a member of the study team will use DocuSign to obtain consent electronically for patients who would like to participate in the trial. They will receive a phone call within 1 week prior to their surgery to confirm the buprenorphine management plan that they were assigned (see randomization and allocation concealment section).

Study staff will contact the provider primarily responsible for the participant’s buprenorphine prescribing at the time of enrollment to confirm their patient’s participation in the study and to review the perioperative buprenorphine dose agreed upon by the participant and the study team. Additionally, study staff will contact the provider 1 month following surgery to determine if the participant is continuing to use buprenorphine, the dose on which they are currently maintained, and if the participant has experienced any episodes of relapse or missed appointments in the intervening post-surgical period.

### 2.4. Randomization and allocation concealment

Enrolled participants will be randomized 1:1 into 1 of 2 groups, stratified according to anticipated pain level POD 1 (see Supplement 1, Supplemental Digital Content, http://links.lww.com/MD/I129). The statistical analyst will provide the research coordinator with randomization assignments in sequentially-numbered opaque envelopes (1 series of envelopes for each stratum of anticipated post-surgical pain). Within each stratum, participants will be randomized to 1 of 2 groups as follows:

- FULL-BUP group: buprenorphine formulation continued throughout the perioperative period.
- STD-BUP group: buprenorphine formulation reduced to 16mg the day prior to surgery and then 8 mg on the day of surgery onwards. Participants in this group taking 12 to 16 mg buprenorphine at baseline will have no change to their buprenorphine dose on the day prior to surgery.

The randomization scheme was developed using NQuery Software (Statistical Solutions, Boston, MA) and uses permuted blocks of 4 and 6 patients to ensure balance between study arms. We used data from Gerbershagen et al,^[20]^ documenting the distribution of pain scores for 179 different surgical procedures, to establish our randomization strata.

All participants will be instructed to resume taking their regular dose of buprenorphine by POD7. This transition will be coordinated between the study team and the participant’s buprenorphine provider. Both patients and providers will be aware of the randomization assignment. However, to help mitigate bias, the study staff collecting and entering data will be masked regarding the patient’s assignment.

### 2.5. Interventions and data collection

Participants will be randomized to receive their full dose of buprenorphine on the day throughout the study period or a reduced dose of 8 mg. Both groups will receive intraoperative and postoperative opioid and non-opioid pain medications as needed based on provider clinical judgement. All analgesic medications administered will be documented by study staff. Intraoperative opioid consumption, type of anesthesia and 24, 48 up to 72-hour opioid consumption will be collected by study staff. All opioid consumption will be converted into MME and buprenorphine formulations will not be included in any calculations of total MME at any timepoint in the study.

**Figure 1.**
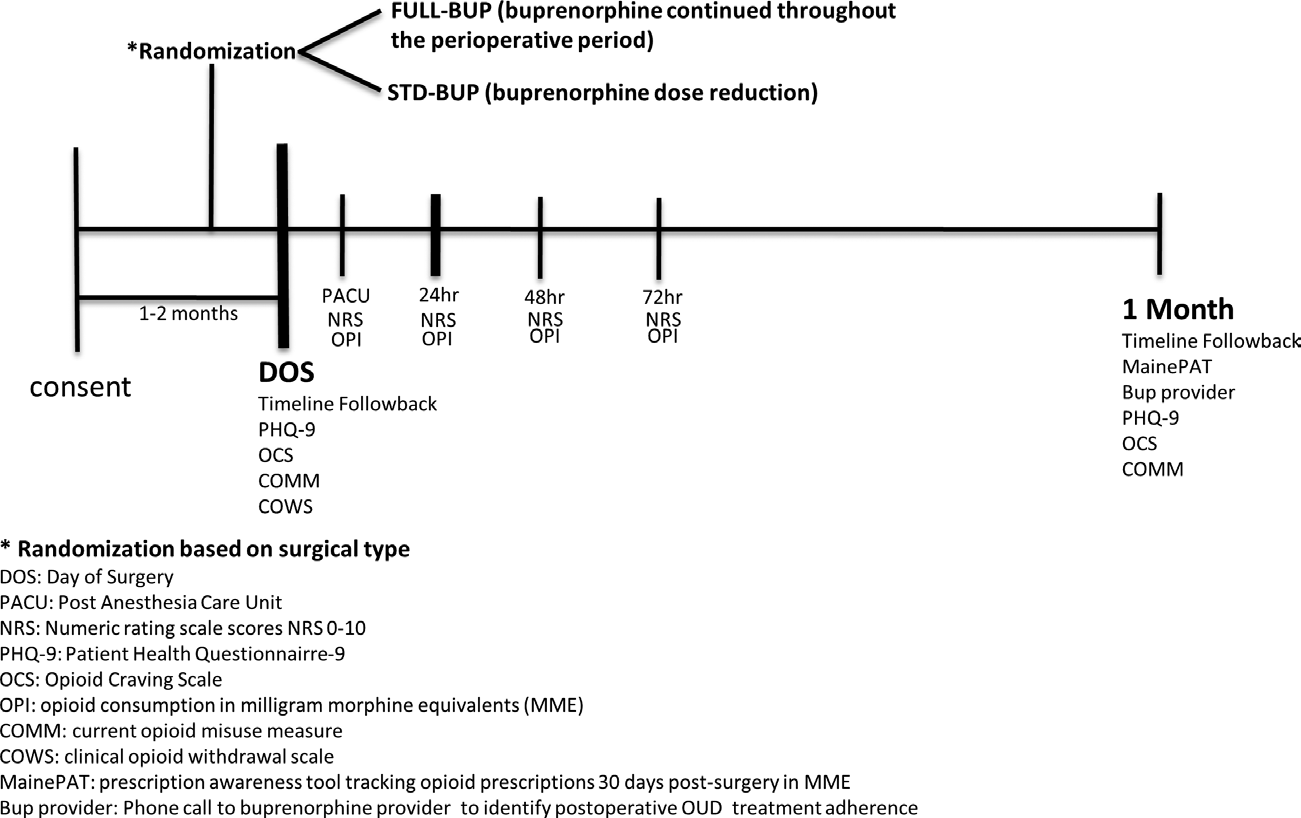
Protocol schema.

On the day of surgery and prior to the surgical procedure, participants will be asked to complete a series of questionnaires to obtain baseline self-assessments for opioid cravings, opioid misuse, mood and opioid withdrawal symptoms (please see Fig. 1 for a schedule of study events). The questionnaires they will be administered are the following:

*PHQ-9 —* a validated self-assessment screening tool used to evaluate mood and depression severity.^[21]^

*COMM* — self-reporting screening tool to measure risk for aberrant opioid medication behavior in patients receiving opioids for pain management.^[22]^

*OCS* — three-item craving measure validated to monitor risk of opioid misuse in patients with OUD.^[23]^

*Clinical opioid withdrawal score* — 11 item scoring assessment administered by a clinician to measure symptoms of opioid withdrawal.^[24]^

Additionally, patients will record pre-operative opioid consumption using a 30-day Timeline Follow-back questionnaire (https://www.smashlabs.org/timeline)^[25]^ to obtain a quantitative estimate of opioid use in the month prior to surgery.

One month postoperatively, participants will receive virtually- administered questionnaires, including: a substance use evaluation and self-report of postoperative pain and opioid consumption using the 30-day Timeline Follow-Back questionnaire. The self-assessments they will complete are identical to those administered on the day of surgery and will be used for comparison analysis.

Study staff will contact each participant’s buprenorphine provider to collect data regarding buprenorphine treatment retention following surgery and will assess each patient’s Maine Prescription Monitoring Program (MainePMP) record to collect data for outpatient opioid utilization during the 30 days following surgery. MainePMP is part of the monitoring program that tracks all schedule II to V medications prescribed in Maine, Massachusetts, and Rhode Island.

### 2.6. Outcome measures

#### 2.6.1. Pain score.

The primary outcome of this study will be average pain scores for each 24-hour period after surgery, up to 72 hours. If a participant is discharged prior to 72 hours after surgery, NRS scores will be assessed at the remaining time points via telephone. Prior to discharge, study staff will provide patients with a log to keep a record of their pain scores, at least once every 8 hours, in order to mitigate recall bias in anticipation of being contacted by research staff. Study staff will call discharged patients and ask them to report their pain scores for each 24 hour period between discharge and 72 hours postoperatively as recorded on the forms they were given at time of discharge.

#### 2.6.2. Opioid consumption during perioperative period.

Opioid consumption data will be collected intraoperatively and over the 24, 48, and 72 hour time periods following surgery. Opioid amounts (excluding buprenorphine) will be converted into MMEs. Patients who are discharged prior to 72 hours after surgery will be given a log to record the pain medications they consume during that period. Study staff will contact patients via telephone to collect this data.

#### 2.6.3. Mood and OUD severity.

To evaluate mood and depression severity, we will use the PHQ-9. For our OUD severity assessments, we will administer the OCS to determine level of opioid cravings, the COMM to detect opioid misuse, and a 30-day Timeline Follow-Back questionnaire to obtain a quantitative estimate of substance use in the month prior to surgery. We will compare the pre-operative and 30-day postoperative participant responses to the above instruments to assess differences between the FULL-BUP and STD–BUP groups. On the day of surgery, we will also the Clinical opioid withdrawal score to detect incidence of opioid withdrawal which can occur with buprenorphine dose reduction or discontinuation.

#### 2.6.4. Opioid dispensing post discharge.

For our assessments on outpatient opioid utilization during the 30 days following surgery, we will review each participant’s MainePMP. Using this resource, we will collect data on the number of opioid agonist prescriptions filled and the total amounts of opioids (in MME) dispensed. Buprenorphine formulations will not be included in our MME totals.

### 2.7. Confidentiality

Participant confidentiality will be protected according to the regulations set forth by the MaineHealth Institutional Review Board IRB and will comply with Health Insurance Portability and Accountability Act regulations. All conversations about their history of OUD and the medications they are taking for treatment will be kept confidential and participating in this study would not cause any additional exposure to their health history outside of standard of care. Participants will be informed that they have the right to not answer any question that makes them feel uncomfortable. Only the study team will have access to research records; paper records will be secured in a locked office and computer data will be encrypted and password protected.

### 2.8. Data management and statistical analysis

Data will be collected and stored in a REDCap database to preserve privacy and confidentiality. The 30-Day Timeline Followback will be completed on Timeline, a website (https://www.smashlabs.org/timeline) developed and supported by Brown University.^[25]^ The responses on the Timeline website will be identified by study numbers only, no PHI will be collected or stored on this site. Information regarding substance abuse history, including illicit narcotic use, will remain confidential and personal identifiers will be removed during data storage. Only members of the research team will have access to the data that participants have consented to provide.

Data will be summarized using descriptive statistics and will be presented as means (SD), medians [IQR] or frequency counts (%), as appropriate. Data will be analyzed on an intention to treat basis. We will examine the success of randomization by comparing demographic and clinical covariate measurements between the FULL-BUP and STD-BUP groups. We will perform univariate analyses to compare outcome measures between the FULL-BUP and STD-BUP groups, using a 2-sided *t* test or Mann–Whitney *U* test for continuous variables and chi square or Fisher exact tests for categorical data, as appropriate. For paired analysis of pre/post surgery instruments, we will use McNemar’s test or the Wilcoxon test, as appropriate. We will explore the relationships between demographic and clinical variables and outcome measures in univariate analyses, using the above methods or Pearson’s and/or Spearman’s correlation, ANOVA or Kruskal-Wallis test, as appropriate. We will explore any observed differences in outcome measures between study arms by adjusting for covariates using analysis of covariance or mixed models regression. Covariates will be entered into the model if they demonstrate a significant (*P* ≤ .05) association with outcome measures in univariate analyses.

We will perform an interim analysis at 50% enrollment and will terminate the study if a significant pain score difference is detected. Significance for the interim analysis will be accepted at *P* < .003, based on 2 sequential tests and using an O’Brien-Fleming spending function to determine test boundaries. Significance overall will be accepted at *P* < .047 to account for interim analysis. All statistical analyses will be performed using SPSS Statistical Software version 28 (IBM SPSS Inc., Armonk, NY).

### 2.9. Sample size

We estimated our study size based on postoperative pain scores in a recent study of surgical patients at our institution; mean NRS on a scale of 0 to 100 was 50 ± 28. Our estimate for effect size is based on published data showing that a difference in NRS of 20/100 points is clinically significant.^[26]^ A sample size of 32 per group will have 80% power to detect a difference in means of 20 (the difference between a Group 1 mean, m1, of 50 and a Group 2 mean, m2, of 30) assuming the common standard deviation is 28 using a 2 group *t* test with a 0.05 significance level. This NRS scale is equivalent to a 0 to 10 scale.^[18]^ To account for dropout, we will enroll an additional 8 patients for each group for a total sample size of 80 patients.

### 2.10. Adverse events and safety

Our study will use a data safety monitoring board (DSMB) that will be scheduled to meet once the study reaches 50% enrollment and at any time in response to an adverse event. The DSMB will review the progress of the study, monitor data, identify any inconsistencies or errors in data collection, and review patient safety. The DSMB for this study is comprised of a group of anesthesiologists, addiction medicine specialists, and a pharmacist. Any errors in data collection that are identified will be reconciled immediately to ensure the validity of our findings. The DSMB will determine study stop rules; however, we will recommend to them that when 10 patients have been enrolled in each group (25% enrollment), if > 7 in either group have intractable pain interfering with hospital discharge or opioid overdose within 2 weeks of discharge, the study should be terminated. The DSMB will also ensure that this study will not continue to enroll patients if 1 treatment modality shows early and significant superiority.

Refer to Table 2 for a list of major and minor adverse events (AEs) that will be reported to the DSMB during the study period and the actions that will be taken. Any unanticipated adverse events will be reported to the IRB within 7 days. For patients who experience AEs related to substance use in the outpatient setting, an addiction specialist on study staff will provide patients with information regarding community resources available to patients, such as support groups and harm reduction resources. Study staff will also contact the patient’s buprenorphine provider to inform them of any AE related to substance use.

**Table 2:** Major and minor adverse events.

	Item	Action	Regulatory reporting
Major AE	1. ED admission due to severe pain 2. Intractable pain	APMS for management recommendations	Immediate to DSMB; within 7 d to IRB
	3. Opioid misuse/relapse 4. Opioid overdose 5. Opioid withdrawal	Alert buprenorphine provider on record immediately; provide patients with community resources to seek help as needed	
Minor AE	1. Hospital discharge delay due to poor pain control 2. Pain worse than anticipated 3. Cravings	Alert IMAT team and APMS as needed	Within 30 d of event to DSMB; DSMB reports to IRB within 7 d

AE = adverse events, APMS = alert acute pain service, DSMB = data safety monitoring board, ED = emergency department, IRB = institutional review board, IMAT = integrated medication assistance treatment.

We will ensure that appropriate plans are in place to guarantee the safety of enrolled subjects. All patients will be made aware of the possible risks associated with treatment group to which they are randomly assigned, as well as the risks inherent in experiencing postoperative pain. Both groups are at risk of experiencing OUD symptom exacerbation. The use of opioid agonists and perioperative pain itself may be risks for OUD symptom exacerbation and relapse. Reduction of buprenorphine to 8 mg before surgery as well as full dose buprenorphine continuation can potentially lead to OUD relapse since exposing patients to opioids with similar properties to the agents they misused in the past as management of their acute post-surgical pain can serve as a trigger for relapse. Another potential risk is difficult post-operative pain control due to buprenorphine continuation since the pharmacokinetics of buprenorphine may interfere with the effectiveness of full opioid agonists used for pain control. However, this risk is not greater than the current risk to patients, since full dose buprenorphine continuation is a practice endorsed by SAMHSA. If patients experience poorly controlled pain, they will receive opioid and non-opioid rescue analgesic agents such as nerve blocks or epidurals when indicated, ketamine infusions, and alpha agonist medications, as determined necessary by the treatment providers caring for the patient. All participants will be transitioned back to their regular dose of buprenorphine when postoperative surgical pain has subsided.

### 2.11. Patient and public involvement

Patients and the general public were not involved in the development or design of this study; including plans for recruitment and overall conduct of the study. The burden of the intervention was not assessed by patients or the public.

### 2.12. Ethics and dissemination

This study was reviewed and approved by the MaineHeath IRB. In the event of any changes to the protocol that impacts currently enrolled participants, the research coordinator will contact them by telephone to communicate those changes and any new or increased associated risks. Additionally, the coordinator will arrange to re-consent the participants if necessary. Any changes to the protocol will be submitted to, and approved by the IRB before being implemented.

After data collection and analysis is complete, this study will be written up for publication in a peer reviewed journal, presented at national conferences, and shared with study participants via email. The de-identified data set for this study will be available upon request and after review and approval by MaineHealth legal team and completion of a data use agreement.

## Author contributions

AQ developed and designed this study, wrote the first draft of this manuscript, and obtained funding. KS, JR, YI, WC, and CR assisted with study design. WC created the statistical plan and randomization scheme for this study. All listed authors assisted with the revision of this manuscript and all approved the submitted version. No professional writers were used.

**Conceptualization:** Aurora Quaye, Kristen Silvia.

**Formal analysis:** Wendy Y. Craig.

**Funding acquisition:** Aurora Quaye, Clifford Rosen.

**Investigation:** Aurora Quaye, Janelle Richard, Yussr Ibrahim.

**Methodology:** Aurora Quaye, Kristen Silvia, Janelle Richard, Yussr Ibrahim, Wendy Y. Craig.

**Project administration:** Janelle Richard.

**Resources:** Kristen Silvia, Clifford Rosen.

**Supervision:** Aurora Quaye, Clifford Rosen.

**Validation:** Janelle Richard.

**Visualization:** Wendy Y. Craig.

**Writing – original draft:** Aurora Quaye, Janelle Richard, Wendy Y. Craig.

**Writing – review & editing:** Aurora Quaye, Kristen Silvia, Janelle Richard, Yussr Ibrahim, Wendy Y. Craig, Clifford Rosen.

## Supplementary Material
