# Supplementary material for: A prospective, randomized trial of the effect of buprenorphine continuation versus dose reduction on pain control and post-operative opioid use
Source: Medicine (Baltimore). 2022 Dec 23;101(51):e32309. doi: 10.1097/MD.0000000000032309 (PMC9794322; doi:10.1097/MD.0000000000032309)
Supplement: Supplementary file 1 [file medi-101-e32309-s001.pdf]

ONLINE SUPPLEMENT 1: Surgical inclusion criteria for elective procedures with greater than 4/10 average pain intensity on postoperative day (POD) 1

Moderate pain score (4-6/10 POD1)

- Closed reduction internal fixation-upper and lower extremity procedures
- Arthroscopic upper and lower extremity joint procedures (i.e. shoulder arthroscopy)
- Clavicle repair/reconstruction
- Hip joint replacement
- Amputation above and below the knee
- Laminectomy
- Spinal canal decompression
- Laparoscopic abdominal surgical procedures including:
  - Appendectomy
  - Cholecystectomy
  - Bowel resection
  - Lysis of adhesions- bowel obstruction
  - Gastrectomy
  - Nissen Fundoplication
- Hernia repair (ventral, inguinal and femoral)
- Resections involving the rectum
- Thyroidectomy
- Nephrectomy (laparoscopic)
- Hysterectomy (vaginal)
- Laparoscopic gynecologic procedures including:
  - Myomectomy
  - Hysterectomy
  - Oophorectomy
  - Endometriosis excision
- Kidney transplantation (laparoscopic)
- Cystectomy
- Prostatectomy
- Orchidectomy
- Femoral and popliteal bypass graft procedures
- Sternotomy procedures
- Thorascopic procedures (i.e. lung wedge resection)
- Breast reconstruction
- Surgical reconstruction nasal septum
- Removal, reconstruction teeth

Severe pain score (>6/10 POD1)

Open Reduction Internal Fixation- upper and lower extremity procedures

Shoulder replacement

Knee joint replacement

Open and arthroscopic foot procedures

Arthrodesis- foot and phalangeal joints

Complex spinal reconstruction, (i.e. scoliosis repair)

Spinal fusion

Laparoscopic procedures converted to open

Nephrectomy (open)

Open thoracotomy procedures

Open gynecologic procedures including:

- Myomectomy
- Hysterectomy

Open abdominal procedures including:

- Pancreatectomy
- Proctocolectomy
- Splenectomy
- Liver resection
- Bowel resection

Adapted from:

Gerbershagen HJ, Aduckathil S, van Wijck AJ, Peelen LM, Kalkman CJ, Meissner W. Pain intensity on the first day after surgery: a prospective cohort study comparing 179 surgical procedures. Anesthesiology. 2013 Apr;118(4):934-44.
