# Supplementary material for: A prospective, randomized trial of the effect of buprenorphine continuation versus dose reduction on pain control and post-operative opioid use
Source: Medicine (Baltimore). 2022 Dec 23;101(51):e32309. doi: 10.1097/MD.0000000000032309 (PMC9794322; doi:10.1097/MD.0000000000032309)
Supplement: Supplementary file 2 [file medi-101-e32309-s002.pdf]

	Human Research Protection Program<br>Institutional Review Board<br>**Consent to Participate in a Research Project and Authorization to to use and Disclose Protected Health Information (PHI)**	

STUDY TITLE: **A Prospective, Randomized Trial of the Effect of Buprenorphine Continuation versus Dose Reduction on Pain Control and Post-Operative Opioid Use**

CONSENT VERSION DATE: 3/11/2022

HOSPITAL OR INSTITUTION: Maine Medical Center

INVESTIGATOR: Aurora Quaye, MD

SUBJECT'S NAME (printed):____

Part I: Key Information About this Research Study

You are being asked to volunteer in a research study. You do not have be in this study. Even if you do agree, you can still leave the study at any time without any penalty, without giving a reason, and will still be able to continue your medical care at MaineHealth. Withdrawal or refusing to be in the study will not affect your relationship with MaineHealth in anyway.

Why is this study being done?
The purpose of this research study is to compare two ways of managing pain in people who are taking buprenorphine and are scheduled to undergo surgery. Buprenorphine (subutex) and buprenorphine/naloxone (suboxone) are effective long-term treatments for substance use disorders. The management of pain after surgery in adults taking buprenorphine can be challenging, as buprenorphine may interfere with the effectiveness of other medications used to treat pain.

We want to compare how well pain is managed after surgery ("post-op") in two groups:

<u>The "Reduce Group":</u> those who are placed on a <u>lower</u> dose of buprenorphine starting one day before surgery and during the time period after surgery until the pain from the surgery has decreased. Once the pain from the surgery has decreased, you will be put back on your full dose of buprenorphine.

The "Continue Group": those who continue to take the full prescribed dose of buprenorphine before surgery, on the day of surgery, and postoperatively.

We also want to find out if there is a difference in pain, opioid cravings, and relapse rates in the month following surgery.

You and the study doctor cannot choose your study group. You will have an equal chance of being assigned to either group. As part of the study for research purposes, on the day of surgery, you will also be given two brief questionnaires about your thoughts and feelings about opioid medications and illicit substances. This will include the questions about cravings and your most recent substance use. Your responses to the questionnaires will be confidential and will not be a part of your medical record. We will ask you to fill out the same questionnaires one month following your surgery to determine if there were any changes to your initial assessment. You will be restarted on the full dose of your medication after adequate healing following your surgery (typically 5-7 days following surgery). We will also contact and notify your buprenorphine provider about your participation in the study and we will ask them questions about your health and your substance use disorder history.

Everyone that signs up for this study will be given the same types of medications to treat pain after surgery. You will be given pain medications whenever you need them for your pain. These medications will be given to you by mouth or through a needle into your veins ("IV"). You will be given opioid medications, such as morphine or oxycodone, and non-opioid medications such as Tylenol and Ibuprofen. The pain medications that we will give you will be the same medications that you would get if you did not participate in the study.

Why are you being asked to be in this research study? You are being asked to take part in this study because you are a healthy adult between the ages of 18-75 that is scheduled to have surgery and you currently are prescribed buprenorphine or buprenorphine/naloxone for the treatment of opioid-use disorder.

How many people will take part in this study and how long will it take? This study will include approximately 80 study participants at Maine Medical Center and it will take, in total, roughly one year to complete the study.

How long will you be in the study and how much time will it take up?
After you are enrolled in the study, you will be in the study for one month.

We will speak with you up to two months before your surgery during your pre-operative assessment to find out if you are interested in taking part in this research study. If you are eligible and agree to take part, we will assign you by chance (like a coin toss) to either continue taking or reduce the dose of your buprenorphine the day before the surgery. You and your study doctor cannot choose your study group. On the day of surgery and the 1st and 2nd days after your surgery, we will visit your hospital room to collect information about your level of pain, your mood and your mental-well being. If you are no longer at the hospital at these time points, we will call you by telephone.

One month after your surgery, we will call you by telephone to again ask you questions about your mental well-being and cravings to substances such as alcohol, pain medications and illicit narcotics. All of these followup sessions will last for about 30 minutes.

What will you be asked to do? If you agree to be in this study, you will be asked to:

- Either continue your buprenorphine at the full prescribed dose or reduce your buprenorphine dose on the day before surgery
- Answer questions about your pain, the amount of opioid medications that you used and questions about your mood and mental well-being on the day of surgery, the 1st and 2nd days after surgery and one month following surgery.

What are the risks or discomforts that are possible from being in this study?
If you are assigned to the 'Reduce' Group or the ‘‘Continue' Group there is risk of opioid cravings and possible relapse. If this does occur, please contact the Maine Medical Center study staff. You will have the option of being increased back to your normal dose of buprenorphine and we will contact the doctor that prescribes your buprenorphine. There is the potential risk that your pain will be difficult to control however there are opioid and non-opioid treatment strategies that can and will be used to treat your pain.

You should also contact study staff or the person that prescribes your buprenorphine at any point in the study if you experience cravings, if your pain is not well controlled, if you have worsening anxiety, restlessness or any other symptoms you think are because of the study.

What benefits to you are possible if you participate in this study?
You will not directly benefit from taking part in this research. We hope that what we learn from this study will help people in the future who are taking buprenorphine/naloxone scheduled for elective surgery.

If you say no to being in this study, do you have other options for your condition?
Your participation in this study is completely voluntary. If you do not take part in this research study, your physicians will decide what the best approach is for managing your buprenorphine before your surgery. Your physician will decide whether to stop or reduce your buprenorphine prior to your surgery and will discuss this with you. Both approaches are used at Maine Medical Center routinely. Some doctors reduce this medication before surgery and some stop it all together. Please talk to your regular doctor about these and other options.

Will being in this study cost you anything?
You or your insurance company will not be charged for any tests or services specifically required by this research study unless the tests or services are clinically indicated or part of your standard treatment. You will still be responsible for the cost of your usual ongoing medical care, including procedures, non-study medications, and tests that your study doctor or regular doctor requires during this study as part of your usual medical care

Will you be paid for being in this study?

You will receive a total maximum of $90 added to a reloadable card for taking part in this study. This includes:

- $10 for completing each daily assessment (on the day of surgery and the 1st and 2nd days after surgery)

- $60 for completing the one-month followup virtual session

MaineHealth has contracted with a company that can process these payments to you. This company may receive your name, date of birth, address, gender, guardianship status, participant identifier, social security number and email in order to process these payments. Payments may be made to you the following ways:

- Reloadable Prepaid Card
- Direct bank deposit
- Paper check by mail

If you select the direct bank deposit option, you will need to provide your bank account information into the Participant Payments Pay Portal. You will receive a separate handout with this consent form that explains how the payments will be made. If you do not want to use this company, please let the study team know and MaineHealth can issue you a paper check. If you have any questions, please ask a member of your study team.

If you were injured or harmed as a result of being in the study, who would pay for the necessary medical treatment and/or hospitalization? Please see Part II regarding details concerning payment for injury or harm.

If you decide to be in the study, the researchers will tell you about any important new information that is learned during the course of this study, which might affect your condition or your willingness to continue participation in this study.

Part II. Additional Information and Details

WHAT ARE THE *(OTHER)* RISKS OF THE STUDY?

Being in more than one research study at the same time, or even at different times, may increase the risks to you. It may also affect the results of the studies. Please let a member of this study team know if you are taking part in another research study.

For more information about risks and side effects, contact Aurora Quaye M.D. at ((207) 200-4061).

WHAT ABOUT CONFIDENTIALITY?

At MaineHealth, we will take steps to help make sure that all the information we get about you is kept confidential. Your name will not be used wherever possible. We will use a code instead. All the study data that we get from you will be kept locked up. The code will be locked up too. If any papers and talks are given about this research, your name will not be used.

We want to make sure that this study is being done correctly and that your rights and welfare are being protected. For this reason, we will share the data we get from you in this study with the study team, the sponsor of the study, MaineHealth's Institutional Review Board, applicable Institutional officials, and certain federal offices, including the Office for Human Research Protections (OHRP), and, where applicable, the Food and Drug Administration (FDA). However, if you tell us you are going to hurt yourself, hurt someone else, or if we believe the safety of a child is at risk, we will have to report this.

This research is covered by a Certificate of Confidentiality from the United States National Institutes of Health (NIH). The researchers with this Certificate may not disclose or use information, documents, or biospecimens that may identify you in any federal, state, or local civil, criminal, administrative, legislative, or other action, suit, or proceeding, or be used as evidence, for example, if there is a court subpoena, unless you have consented for this use. Information, documents, or biospecimens protected by this Certificate cannot be disclosed to anyone else who is not connected with the research except, if there is a federal, state, or local law that requires disclosure (such as to report child abuse or communicable diseases but not for federal, state, or local civil, criminal, administrative, legislative, or other proceedings, see below); if you have consented to the disclosure, including for your medical treatment; or if it is used for other scientific research, as allowed by federal regulations protecting research subjects.

The Certificate cannot be used to refuse a request for information from personnel of the United States federal or state government agency sponsoring the project that is needed for auditing or program evaluation by the NIH which is funding this project or for information that must be disclosed in order to meet the requirements of the federal Food and Drug Administration (FDA). You should understand that a Certificate of Confidentiality does not prevent you from voluntarily releasing information about yourself or your involvement in this research. If you want your research information released to an insurer, medical care provider, or any other person not connected with the research, you must provide consent to allow the researchers to release it.

The Certificate of Confidentiality will not be used to prevent disclosure as required by federal, state, or local law of harm to yourself or others

A description of this research study will be available on http://www.Clinicaltrials.gov, as required by U.S. Law. This website will not include information that can identify you. At most, the website will include a summary of the results. You can search this website at any time.

IF I AM BEING COMPENSATED FOR BEING IN THIS STUDY
Please check the appropriate box:

__ You are a U.S. Citizen or Resident Alien. If you are paid $600 or more a year from MaineHealth, your social security number and amount paid will be reported to those in charge of taxes (IRS) and you may have to pay taxes on this money.

__ You are a Nonresident Alien. For tax purposes, all payments made to you, including those for your participation in this study, are subject to a 30% tax withholding. All withholdings and payments will be reported to those in charge of taxes (IRS) by MaineHealth.

___ If you do not wish to be paid for your participation in this study, please initial here: ____

WHAT IF I AM INJURED DUE TO MY PARTICIPATION IN THIS STUDY?

In the case of injury or illness resulting from this research study, medical treatment will be available at the usual charge.

MaineHealth has no policy or plan to pay for any injuries that you might receive as a result of your participation in this study. However, this does not take away your rights to seek or collect compensation for injury related to malpractice, fault, or blame on the part of those involved in the research, including the hospital.

You or your insurance company will be responsible for any costs resulting from underlying disease or treatments provided to you outside of this research study.

HOW CAN I WITHDRAW FROM THIS STUDY?

You can always choose to stop participating in this study. If you decide that you want to change how your buprenorphine dose is being managed and you no longer want to be a part of the study, you can let us know that you no longer want to participate in the study and you can discuss with your physician the best approach for managing your buprenorphine. Your physician would then decide how to manage your buprenorphine and will discuss this with you. If you are experiencing cravings, relapse or worsening anxiety or depression that you feel is due to your involvement in the study, please contact Aurora Quaye, MD and we can remove you from the study and provide you with any resources that you need to improve your symptoms.

The information collected prior to your withdrawal from the study will continue to be part of the data and included in the final analysis for this research. No new information will be collected. All records that link your identity to the research information will be destroyed. The anonymous information will continue to be part of the research data.

Removal from Study

The researcher may require that you be withdrawn from participation. This may happen if you are experiencing severe cravings, relapse or mental health concerns that we deem would lead to your physical harm. Although you would no longer be enrolled in the study, we would provide you with the resources and connect you with providers that would help you remain safe.

HOW WILL MY INFORMATION/SAMPLES BE SHARED?

Information obtained during this study may become the property of MaineHealth. When your involvement with the study ends, you may be asked to participate in a research registry/repository (i.e. a research bank). Participation in the research bank would allow for storage of your identifiable information and would also allow the manager of the research bank

to contact you about potential participation in future studies that would like to use your identifiable information. The manager of the research bank could also de-identify your information and share it with other investigators without additional consent from you. You will be asked to provide separate consent for participation in the bank and may decline.

We receive money from the National Institutes of Health (NIH) to do this study. NIH requires that we have a plan in place to share information we gain in this study. We anticipate publishing the findings of this research and publishers often require that we have a plan in place to share the information we collect during this study.

Your information will only be shared in an anonymous way. Sharing research data helps to translate research results into knowledge, products, and procedures that improve human health. If you provide permission now to share your anonymized information with the database noted below, you may withdraw your permission later without any penalty or loss of benefit. The information will be withdrawn from the database. However, if the information has already been shared with other researchers that information will not be able to be deleted.

Using your information and tissues from this study for future research:
Research using data and tissues is an important way to study and understand human disease.

Identifiers might be removed from your study information or tissues and could then be used for future research studies or distributed to another investigator for future research studies without additional informed consent from you (or your legally authorized representative)

Permission for the research team to obtain and use your patient health information

How will the privacy of my patient health information be protected?

There are state and federal privacy laws that protect the use and sharing of your patient health information. By signing this form, you provide your permission, called your "authorization," for the use and sharing of patient health information protected by the Privacy Rule. Authorization includes allowing:

- Your health care providers to share your health information for this research study
- The research team to use and share your health information for this research study.

Health information about you that will be used or shared with others involved in this study may include your research record and any health care records at MaineHealth. **Specifically, this will include your name, mailing address, medical record number, social security number, date of birth, gender, guardianship status, participant identifier, date of service, substance use history, mental health information, phone number,and email address**. This may also include any new health information about you that comes from the

research tests or procedures described in this consent form. Psychotherapy notes in your health records (if any) will not, however, be shared or used. Use of these notes requires a separate, signed authorization.

The research team and people within MaineHealth who oversee and help administer research may see, use or share your information as needed for the research.

People outside of MaineHealth may need to see or receive your information for this study. Examples include government agencies (such as the Food and Drug Administration), safety monitors, other sites in the study and companies that sponsor the study.

We cannot do this study without your authorization to use and share your information. You do not have to give us this authorization. If you do not, then you may not join this study.

We will use and share your information only as described in this form; however, people outside MaineHealth who receive your information may not be covered by this promise or by the federal Privacy Rule. We try to make sure that everyone who needs to see your information keeps it confidential – but we cannot guarantee that your information will not be re-disclosed.

The use and sharing of your information has no time limit. You may revoke (cancel) your permission to use and share your information at any time by notifying the Principal Investigator of this study by phone or in writing. If you contact the Principal Investigator by phone, you must follow-up with a written request that includes the study number and your contact information. Send your request to: **Aurora Quaye, M.D., 22 Bramhall St., Portland, ME 04102 attn: Aurora Quaye, MD**

If you do cancel your authorization to use and share your information, your part in this study will end and no further information about you will be collected. Your revocation (cancellation) would not affect information already collected in the study, or information we shared before you wrote to the Principal Investigator to cancel your authorization.

Your decision to not sign this authorization will not affect any other treatment, health care, enrollment in health plans or eligibility for benefits.

If this study is related to your medical care, your study-related information may be placed in your permanent hospital, clinic, or physician's office records at MaineHealth.

Finally, with your initials, please specifically authorize the use of your private health information relating substance abuse, mental health information, or HIV/AIDS, if applicable, for the above-described purposes.
Initials: ________________

WHOM DO I CALL IF I HAVE QUESTIONS OR PROBLEMS?
For questions about the study or a research-related injury contact Aurora Quaye, MD at (207) 200-4061.

For questions about your rights as a research participant, or to provide input, contact the MaineHealth Institutional Review Board (which is a group of people who review the research to protect your rights) at (207) 661-4474. Alternatively, you may provide comments or ask questions in the Human Research Protection Program Feedback section on our website at http://mmcri.org/ns/?page_id=17782 .

I have read, or have had read to me, the above information before signing this consent form. I agree to take part in this research study. I also give permission to use or share my personal health information for the purpose of this research. I have had the chance to ask questions. I have received answers that fully satisfy those questions.

_______________________________ _______________
Signature of Subject Date

____
Printed Name of Subject

<u>Statement of Witness to Consent of Subjects Who Cannot Read or Write</u>

I represent that the consent form was presented orally to the subject in the subject's own language, that the subject was given the opportunity to ask questions, and that the subject has indicted his/her consent and authorization for participation (check one box as applicable):

- ☐ Making his/her mark above
- ☐ Other means ____
  (fill in above)

_______________________________ _______________
Witness for adults unable to read or write Date

Study representative statement
I have fully explained in terms understandable to the subject all of the following: the purpose of this research, the study procedures, the possible risks and discomforts and the possible benefits. I have answered all of the subjects and his/her authorized representative(s) question to the best of my ability. I will inform the subject of any changes in the procedure or the risks and benefits if any should occur during or after the course of the study.

_______________________________ _______________
Signature of the Person Obtaining Consent Date

____
Printed Name of the Person Obtaining Consent

A signed copy of this consent form must be given to each subject entering the study.
